# Medical Society of the State of New York

**Published:** 1891-03

**Authors:** 


### BUFFALO MEDICAL AND SURGICAL JOURNAL

A MONTHLY REVIEW OF MEDICINE AND SURGERY.

*EDITORS:*

THOMAS LOTHROP, M. D. - - WM. WARREN POTTER, M. D.

All communications, whether of a literary or business character, should be addressed to the editors : 284 FRANKLIN STREET, BUFFALO, N. Y.

VOL. XXX. MARCH, 1891. No. 8.

### Editorial.

### MEDICAL SOCIETY OF THE STATE OF NEW YORK.

THE eighty-fifth annual meeting of this Society, held in Albany, February 3d, 4th, and 5th, was both notable and interesting. The attendance was large, the scientific work excellent, and harmony was manifest on all sides. There were present distinguished guests from several states and Canada, who participated in the proceedings, giving additional interest to the meeting.

Drs. L. S. McMurtry, of Louisville, and Fayette Dunlap, of Danville, represented Kentucky; Dr. W. E. B. Davis, of Birmingham, represented Alabama ; Dr. Charles A. L. Reed, of Cincinnati, represented Ohio ; Drs. E. L. Shurly, of Detroit, and Heneage Gibbes, of Ann Arbor, represented Michigan ; Dr. Francis Bacon, of New Haven, represented Connecticut; Drs. A. H. Wright and Jas. F. W. Ross, of Toronto, represented Canada; Drs. W. W. Keen and E. E. Montgomery, of Philadelphia, represented Pennsylvania.

The discussion on Tuesday evening upon Appendicitis was a brilliant surgical symposium, in which eight or ten of the prominent surgeons both from within and without the State participated.

A special interest centered around the fact that at this meeting the first State Medical Examining and Licensing Board was to be nominated. It was apprehended that the selection of the names to compose this Board would lead to discussion that might occupy time properly belonging to the scientific work, hence it became a matter of considerable importance to so dispose of this great duty as to properly represent the dignity of the Society as well as economize time. A representative committee was happily chosen to nominate fourteen names to the Regents of the University, seven of whom are to compose the Board, and it is difficult to see how this committee could have performed its duties in a more satisfactory manner. Certainly the committee should feel great satisfaction in having its work unanimously indorsed by the Society. These nominations are as follows: Dr. W. C. Wey, Dr. B. F. Sherman, Dr. W. W. Potter, Dr. W. S. Ely, Dr. Geo. F. Shrady, Dr. J. P. Creveling, Dr. L. S. Pilcher, Dr. Edward B. Angell, Dr. Geo. R. Fowler, Dr. H. D. V. Pratt, Dr. C. L. Dana, Dr. Eugene Beach, Dr. V. P. Gibney, and Dr. M. J. Lewi. The several parts of the State have been geographically considered in the selection of these names, and the interests of the Society as well as the profession at large seemed to have been well conserved thereby.

The social features of the meeting were unusually pleasant, and deserve to be mentioned. Dr. Vander Veer's dinner to the Ex-Presidents of the Society on Monday evening, at the Fort Orange Club, and to which out-of-the-State guests were also invited, was a fitting commencement to the meeting, that will be remembered as one of the best in the history of the Society. A number of private dinners followed on Tuesday evening, and the Society's banquet on Wednesday evening was a suitable *finale* to the unofficial part of the gathering. At the latter two hundred and fourteen covers were laid, and the proprietor of the Delavan, Mr. Roessle, pronounced it the largest medical banquet ever given in Albany.

The Speaker of the Assemby, Honorable Wm. F. Sheehan, graced the banquet with his presence, and made a witty and eloquent speech that won the plandits of the banqueters.

The officers chosen for the ensuing year are: President, Dr. A. Walter Suiter, of Herkimer; Vice-President, Dr. W. W. Crandall, of Allegany; Secretary, Dr. F. C. Curtis, of Albany; Treasurer, Dr. C. H. Porter, of Albany. Dr. H. R. Hopkins, of Buffalo, was retained on the Committee of Ethics, Dr. Edward Clark, of Buffalo, was appointed a member of the Committee on Hygiene, and Dr. A. E. Persons, also of Buffalo, on the Committee of Publication.

NOTES AND ECHOES OF THE MEETING.

The Special Committee to nominate the State Examining and Licensing Board were: Dr. D. B. St. John Roosa, Chairman; Dr. John O. Roe, Secretary; Dr. B. F. Sherman, Dr. Henry Flood and

Dr. D. V. O'Leary. After two days' hard work in considering this subject in all its phases, the Committee reported at five o'clock, P. M., Wednesday, and the Society unanimously confirmed the names presented. The Society is to be congratulated on the work of this Committee, and the latter deserves the thanks of the Society as well as of the profession at large.

The annual banquet of the Society, on Wednesday evening, at the Delavan House, was a picturesque and delightful grouping of physicians, clergymen, lawyers and legislators. The speeches were bright, brief and pointed, while the sallies of wit were refreshing and convulsing. Mr. Theodore Bacon, of Rochester, who was present by incident of travel, spoke for the lawyers, and rarely has a more entertaining speech been heard on such an occasion.

The Business Committee deserves great credit for its management of the scientific work, which falls to its lot under the by-laws. Rarely has so much material been disposed of in the time allotted, and with so little friction, or expressed satisfaction. This committee was complimented on all sides for its efficiency and affability.

The registration was large — nearly 300 members, delegates, and guests —and the Committee on Credentials was kept busy early and late. The plan of opening the register on Monday evening seemed to meet with favor, enabling the early arrivals to record themselves before the rush of Tuesday morning. Dr. C. W. Hamlin, of Middleville, chairman of this committee, was always at his post, and the exchequer of the Society was largely increased by his watchful care.

The Committee of Arrangements performed its work well, and everything was done to make the labor of the presiding officer easy and pleasant. This means a great deal, for it is within the province of this committee to aid greatly in the administration of the affairs of the Society during the meeting, besides the various other duties that devolve upon it. The genial chairman, Dr. S. B. Ward, was at his best during the banquet, and contributed largely to its éclat.

In a leader, the *Canadian Practitioner*, February 16, 1891, speaks pleasantly of the meeting in several particulars :

It is one of the oldest medical associations on this continent, this being the eighty-fifth annual meeting. Its organization is complete in all respects. Representatives from the various county societies are regularly elected, and generally make it a point to attend the meetings. Certain powers are given to the Society by the State Legislature, corresponding to a certain extent with those given to the Ontario Medical Council by our Government.

The Society is showing a commendable desire to raise the standard of medical education in New York, and through its influence an act has been passed by the State Legislature authorizing the establishment of a central examining board. . . . . . . In comparing it with the meetings of the Ontario Medical Association, we noticed that the proceedings were pushed along at almost railroad speed. For instance, the first morning session commenced at 9.15 o'clock, and its programme included the President's inaugural address, appointments of committees, executive business, and eleven papers. It will probably be a surprise to members of Canadian associations, who see practically nothing done on the first mornings, to learn that this programme was completed. . . . . .

All questions referring to matters not connected with the reading of papers were referred to committees, and much valuable time was saved thereby.

After referring to the banquet, our contemporary concludes as follows:

The after-dinner speeches on this occasion were remarkably good, being, as a rule, bright, witty, and brief. Among those who shone in this particular line were the genial and popular President; two of the most gifted sons of the sunny south, Dr. McMurtry, of Louisville, Kentucky, and Dr. Davis, of Birmingham, Alabama; Dr. St. John Roosa, of New York; Dr. Reed, of Cincinnati; and representatives of the local clergy.

The members extended to their guests a generous hospitality. The visitors from Canada, Drs. J. F. W. Ross and A. H. Wright, of Toronto, were entertained in royal style by the President and the good people of Albany.

The *Boston Medical and Surgical Journal* devotes generous space to the proceedings of the meeting, its account thereof running through several consecutive numbers.

The *Philadelphia Medical and Surgical Reporter*, in its New York correspondence, publishes a lengthy *résumé* of the proceedings, and other journals out of the State have published some of the papers read at the meeting. Among the latter may be mentioned the *Virginia Medical Monthly*, and the *Medical Mirror*.

Dr. William H. Taylor, of this city, was elected Honorary Member of the New York Medical Society, at its late meeting. We are glad to see honors of this character coming from the East. In this instance it is not only complimentary, but most worthily bestowed. —*Cincinnati Lancet-Clinic.*

